# Natural products in the predatory defence of the filamentous fungal pathogen *Aspergillus fumigatus*

**DOI:** 10.3762/bjoc.17.124

**Published:** 2021-07-28

**Authors:** Jana M Boysen, Nauman Saeed, Falk Hillmann

**Affiliations:** 1 Junior Research Group Evolution of Microbial Interactions, Leibniz-Institute for Natural Product Research and Infection Biology – Hans Knöll Institute (HKI), Beutenbergstr. 11a, 07745 Jena, Germany; 2 Institute of Microbiology, Friedrich Schiller University Jena, Jena, Germany

**Keywords:** amoeba predation, *Aspergillus fumigatus*, fungal ecology, non-ribosomal peptides, polyketides, secondary metabolism, virulence

## Abstract

The kingdom of fungi comprises a large and highly diverse group of organisms that thrive in diverse natural environments. One factor to successfully confront challenges in their natural habitats is the capability to synthesize defensive secondary metabolites. The genetic potential for the production of secondary metabolites in fungi is high and numerous potential secondary metabolite gene clusters have been identified in sequenced fungal genomes. Their production may well be regulated by specific ecological conditions, such as the presence of microbial competitors, symbionts or predators. Here we exemplarily summarize our current knowledge on identified secondary metabolites of the pathogenic fungus *Aspergillus fumigatus* and their defensive function against (microbial) predators.

## Introduction

**Figure 1:**
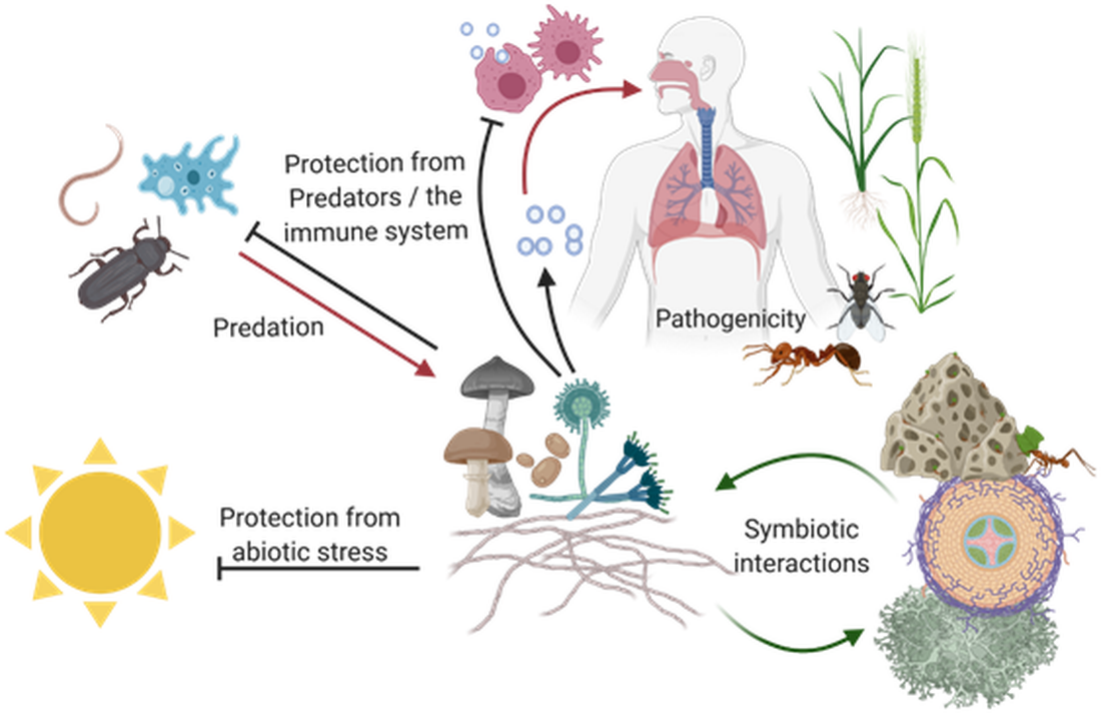
Schematic overview of fungal interactions in the environment. Fungi can be found in essentially all terrestrial habitats comprising saprophytic, parasitic, symbiotic or predatory lifestyles [6]. Independent of their ecological niche, they have to protect themselves against abiotic stresses, competitors and predators while also communicating with their host or partners during parasitic/pathogenic or symbiotic interactions. This figure was created with biorender.com.

To thrive in their natural habitats all organisms from bacteria and fungi to plants and animals need access to sufficient nutritional sources and have to defend themselves against both, competitors and predators (Figure 1). Fungi are ubiquitous, living a mostly saprophytic, parasitic or symbiotic lifestyle in various habitats including soil, water, other organisms and even salt-flats and arctic glaciers [1–2]. As fungi are not able to physically leave their habitats they must rely on mechanical barriers, physiological adaptations and chemical defence mechanisms to optimize their living conditions and resist competitors, parasites and predators [3–5]. These bioactive compounds are often considered as secondary metabolites (SM) which are involved in communication, symbiotic interactions, pathogenicity or chemical defence, e.g., by toxin production [6]. With penicillin as the prime example fungal secondary metabolites have raised scientific and pharmaceutical interests for nearly one century. Today’s sequencing and bioinformatic analyses of fungal genomes revealed that the genetic potential far exceeds the number of known metabolites and the interest of scientists to gain access to them remains high [7–9].

Genes associated with these bioactive compounds are often organized in biosynthetic gene clusters (BGCs) which are physically linked, commonly regulated and often belong to a few distinct classes of molecules like non-ribosomal peptides (NRP), polyketides (PK), terpenes or indole alkaloids [10–11]. The vast majority of fungal BGCs is found in the genomes of members of the Basidiomycota and Ascomycota including the genus *Penicillium* in which the first BGC was identified in 1990 [12–14]. *Penicillium* species belong to the Pezizomycotina, a subdivision within the Ascomycotina including several species that are closely associated with humans at many different levels. Aside from being a source of many medically relevant compounds including antibiotics like penicillin they offer food sources in the form of naturally grown truffles (e.g., *Tuber melanosporum*) or recently cultivated meat alternatives like Quorn^®^ (*Fusarium venenatum*) [15–17]. Species of *Aspergillus*, such as *Aspergillus fumigatus, Aspergillus flavus* and *Aspergillus niger* can affect the health of humans, plants and lifestock by acting as pathogens. It is firmly established that the ability to produce mycotoxins contributes to the virulence potential of these fungi, but as they all thrive in environmental reservoirs they must also provide an ecological advantage to their producer [18].

**Figure 2:**
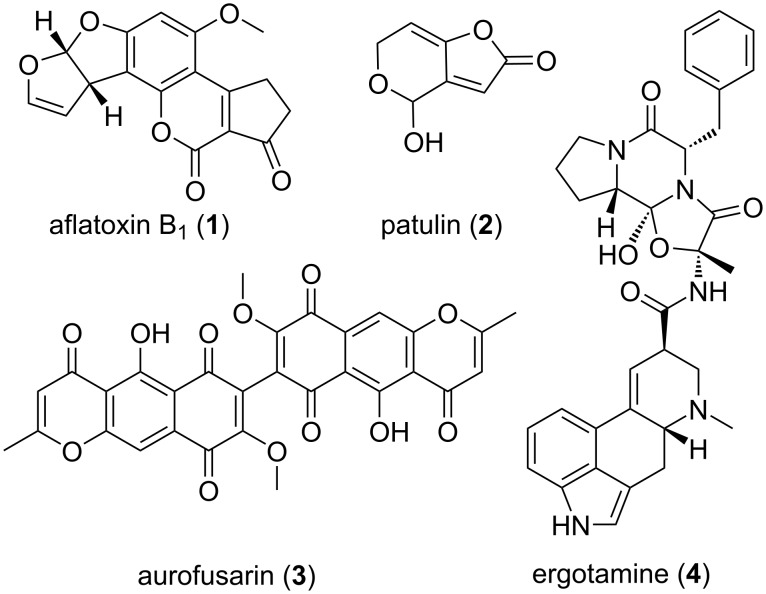
Fungal derived bioactive natural compounds with ecological and/or economic relevance.

Indeed, many of these pathogenic fungi also produce compounds with antibacterial, antifungal and insecticidal properties to ward of both competitors and predators. The mycotoxins aflatoxin B1 (**1**) from *Aspergillus flavus* and patulin (**2**), produced by *Aspergillus* and *Penicillium* species, exhibit insecticidal activity against *Drosophila melanogaster* and might thus prevent feeding competition [19–21] (Figure 2). But not only mycotoxins protect from predation: *A. flavus* sclerotia are protected from sap beetles by asparasone and *Neurospora crassa’s* neurosporin A prevents springtail grazing [22–23]. Grazing by *Folsomia candida* springtails on *Fusarium graminearum* induces several metabolites, of which especially the bisnaphthopyrone pigment aurofusarin (**3**) was shown to have antifeedant effects not only on springtails but also on mealworm *Tenebrio molitor* and woodlouse *Trichorhina tomentosa*. Not only *Fusarium* species produce bisnaphthopyrones like aurofusarin but also *Aspergillus* and *Penicilllium* species produce these metabolites which show antifeedant effects on a wide variety of arthropods [24].

Some fungal compounds can have deleterious effects on humans, livestock or crops, like the ergot alkaloids, e.g., ergotamine (**4**) present in the sclerotia of the ergot fungus *Claviceps purpurea*, which can contaminate grain products like flour. In the middle ages these contaminations caused vast epidemics of “St. Anthony’s fire”, a severe poisoning which could lead to death and mutilation in humans. However, midwives already knew the therapeutic potential of ergot alkaloids as early as 1582 and used it for abortion or to aid childbirth. The ecological significance of ergot alkaloids remains unclear, but they are assumed to be a feeding deterrent due to their toxicity and bad taste [25–28].

To trigger the synthesis of new SMs a number of approaches have been exploited so far, including co-cultivation with other species [9]. Amoebae offer promising possibilities to not only discover new SM but also to discover their ecological role as amoeba often cohabitate with fungi in their natural environments, especially the soil. Some, like *Protostelium aurantium*, were recently found to be exclusively fungivorous, feeding on both yeasts and filamentous fungi alike [29]. Additionally, amoeba closely resemble human phagocytic cells and the interactions of fungi and amoeba often parallels interactions of fungi and macrophages as was shown for *Aspergillus fumigatus* and its interactions with *Acanthamoeba castellanii* [30]. Thus, the adaptations that protect fungi against amoeba that were gained in the ‘environmental school of virulence’ might also protect fungi from the immune system [31]. Therefore, to study their interactions with human pathogenic fungi like *A. fumigatus*, one of the most common airborne fungal pathogens, might lead to new insight in virulence mechanisms and the role of SMs therein [32]. The aim of this review is to depict the fungal secondary metabolite potential and its role in an ecological context using *A. fumigatus* as an example because of its high medical importance and its diverse profile of secondary metabolites which seems to fulfil dual roles: targeting innate immune cells during virulence and protect from environmental predators in natural habitats.

## Review

### Natural products of Aspergillus fumigatus

The genus *Aspergillus* comprises a large number of species that are not only of scientific but also of pharmaceutical and commercial interest. While the non-pathogenic *A. niger* is used as industrial workhorse, for example in the production of citric acid, other representatives contaminate food stocks with mycotoxins (*A. flavus*) or can cause severe infections (*A. fumigatus*, *A. terreus*). Despite their different role for humans, they commonly share a high potential for the production of secondary metabolites, measured by the predicted number of secondary metabolite gene clusters identified by numerous genome sequencing projects. Due to its clinical importance as an opportunistic pathogen *A. fumigatus* is of great interest among them [33–34].

As a saprophytic decomposer of organic material in the soil, *A. fumigatus* encounters not only numerous competitors but also fungivorous predators like amoebae (e.g., *P. aurantium*), nematodes (e.g., *Aphelenchus avenae*) or arthropods like insects, mites and springtails (e.g., *F. candida*) [35–39]. However, the fungus may also act as a pathogen causing often lethal infections in immune-compromised patients, and thus its secondary metabolism was extensively studied in recent years [38,40–41]. Analysis of the *A. fumigatus* genome sequence and metabolomics revealed its potential to synthesize more than 200 compounds and the presence of over 30 secondary metabolite associated gene clusters [7,42–44]. The products of many of those gene clusters are already known and span the whole range of secondary metabolite classes. Table 1 provides an overview of the major secondary metabolites from *A. fumigatus* and lists their ecological roles as well as their impact on virulence.

**Table 1:** Overview of *Aspergillus fumigatus* secondary metabolites and their roles during virulence and in their ecological context.

Metabolite	Class	Virulence factor	Role in virulence	Ecological role/ toxicity	Reference

DHN-melanin	polyketide, phenolic polymer, pigment	yes	- prevents recognition by the immune system - prevents phagosomal acidification	- protection against UV-stress - prevents recognition by predators (e.g., amoeba) - prevents phagolysosome maturation	[45–50]
endocrocin	polyketide, pigment	–	- inhibits chemotaxis of neutrophils	- protection against UV-stress	[47,51–53]
ferricrocin	siderophore	yes	- iron homeostasis	- iron homeostasis	[54–55]
fumagillin	mero-terpenoid	–	- inhibitor of phagocyte activity - damages epithelial cells - inhibitor of methionine aminopeptidase	- cilioinhibitory - antimicrobial - antiprotozoal	[56–62]
fumigaclavine	ergot alkaloid	–	- reduces production of TNF-α – toxic to mammalian cells	- antibacterial - insecticidal - antifeedant	[63–67]
fumipyrrole	non-ribosomal peptide	–	–	- enhances growth and sporulation	[68]
fumiquinozalines	tryptophan derived peptidyl alkaloid	–	not determined	- antibacterial - antifungal	[69–72]
fumisoquin	isoquinolone alkaloid	–	not determined	- inhibits bacterial replication	[73–74]
fumitremorgin	indole diketo-piperazine alkaloid	–	- inhibitor of breast cancer resistance protein	- antifungal - antifeedant - insecticidal	[72,75]
fusarinine C/ triacetylfusarinine C	siderophore	yes	- iron acquisition	- iron acquisition	[54–55,76]
fungisporin	non-ribosomal peptide	–	not determined	- antibacterial	[41,77]
gliotoxin	epipolythiopiperazine	yes	- inhibition of immune response	- cilioinhibitory - antimicrobial - protects against amoeba predation	[78–82]
helvolic acid/ protostadienol	fusidane-type steroid	–	- cilioinhibitory	- antibacterial - antiprotozoal - antifungal	[72,83–88]
hexadehydro- astechrome	non-ribosomal peptide, tryptophan-derived iron(III) complex	yes	- iron homeostasis	- iron homeostasis	[89–90]
neosartoricin/ fumicycline	prenylated polyketide, meroterpenoid	–	- inhibition of immune response	not determined	[41,91–92]
nidulanin A	tetracyclo-peptide/ isoprene	–	not determined	not determined	[93]
pseurotin	heterocyclic γ-lactam	–	- inhibition of IgE production	- antibacterial	[94–97]
pyripyropene A	sesqui-terpenoid	–	- acetyltransferase inhibitor	- nematicide - insecticidal	[98–100]
sphingofungin A–D	sphingosine-like compound	not determined	- inhibition of serine palmitoyl transferase	- antifungal	[101–104]
trypacidin	polyketide, anthraquinone, pigment	–	- toxic to lung cells	- antiprotozoal - antiphagocytic	[53,105–107]
verruculogen	indole diketo-piperazine alkaloid	not determined	- alters electrophysical properties of human nasal epithelial cells	- antifungal	[72,108–110]
xanthocillin	tyrosine-derived isocyanide	–	- copper homeostasis	- copper homeostasis	[111]

#### Gliotoxin

**Figure 3:**
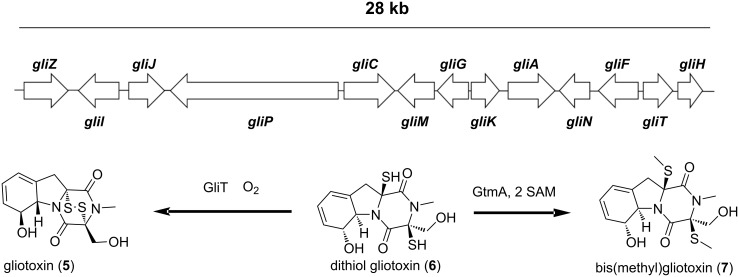
Gliotoxin biosynthetic gene cluster and it major biosynthetic transformations: Gliotoxin (**5**) is the oxidized form of dithiol-gliotoxin (**6**) catalysed by the gliotoxin oxidoreductase GliT. Dithiol-gliotoxin can be methylated to bis(methyl)gliotoxin (**7**) via the S-adenosyl-methionine (SAM) dependent bisthiomethyltransferase GtmA which is not part of the *gli-*cluster.

Gliotoxin (GT, **5**) is the non-ribosomal peptide (NRP) derived epipolythiodioxopiperazine (ETP’s) class toxin of several fungal genera including *Aspergillus*, *Penicillium*, *Trichoderma*, and *Leptosphaera* (Figure 3) [112]. Among the ascomycetes, *A. fumigatus* may well be the major GT producer and the identification of its heterocyclic structure by Bell and colleagues in 1958 builds the foundation to understand its role in invasive aspergillosis [113]. In *A. fumigatus* 13 genes form a 28 kb biosynthetic cluster of gliotoxin, of which *gliZ* (a zinc-finger transcription factor) and *gliP* (an NRPS) together with global regulator LaeA regulate its expression at the genomic level [112,114–116] (Figure 3). Whereas GliT (a gliotoxin oxidoreductase) catalyses the oxidation of reactive dithiol gliotoxin (**6**) to gliotoxin and a distantly localized *S*-adenosylmethionine-dependent gliotoxin bisthiomethyltransferase (*GtmA*) is responsible for the formation of bis(methyl)gliotoxin (**7**) to maintain the GT concentration at sub-lethal levels via redox cycling and S-methylation of active disulfides in GT, respectively [117–118]. Furthermore, in terms of exogenous factors, not only GT itself but several other biotic and abiotic factors, including neutrophilic granulocytes, media composition, pH, temperature and aeration, are known to regulate gliotoxin biosynthesis [115,119–120].

The biological activity of ETP’s like gliotoxin is mediated by the active disulfide bridge that targets vulnerable thiols or catalyses oxidative burst formation via redox cycling [78]. In previous studies, these cytotoxic activities of gliotoxin were shown to be immunosuppressive in humans [79–81]. Sugui and colleagues (2007) also demonstrated that a gliotoxin lacking strain of *A. fumigatus* is avirulent in mice treated with cortisone acetate [121]. Nevertheless, the fact that gliotoxin is not only produced by pathogenic *A. fumigatus* suggests a role of gliotoxin in natural microenvironments. In vitro studies have also revealed the amoebicidal activities of gliotoxin on its natural co-inhabitant *Dictyostelium discoideum* [82]. However, these pathogenic activities sometimes prove to be beneficial for other co-habitants, comparable to how *Trichoderma virens* protects cotton seedlings from its pathogen *Pythium ultimum* [122].

#### Trypacidin

**Figure 4:**
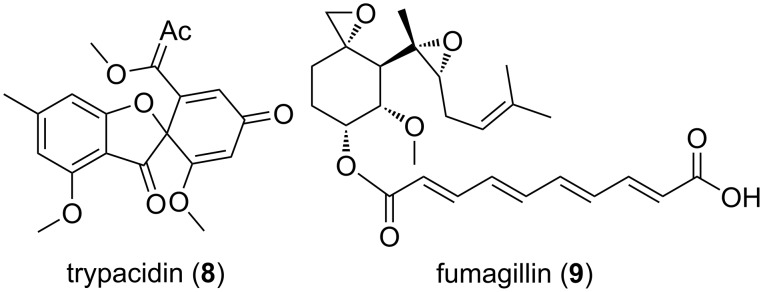
Amoebicidal secondary metabolites trypacidin and fumagillin of *Aspergillus fumigatus*.

The spore-born toxin trypacidin (**8**) is a polyketide that belongs to an anthraquinone-derived class of secondary metabolites (Figure 4) [107]. In *A. fumigatus*, the trypacidin biosynthetic cluster (*tpc*) is comprised of 13 genes that spans over a 25 kb sub-telomeric region on chromosome 4 [53,105]. It is one of the conidial secondary metabolites that are regulated by global transcriptional regulators LaeA and BrlA in *A. fumigatus* [51,123–126]. Nevertheless, trypacidin production is also regulated by cluster specific transcriptional regulators TpcD/E [53]. Though the precise mechanism of action of trypacidin remains to be elucidated, it was shown to exhibit antiprotozoal, antiphagocytic and cytotoxic activities in vitro. Gauthier and colleagues (2012) have shown that in lung cells trypacidin mediates in necrosis-mediated death [107]. In another study, absence of trypacidin was shown to be linked with increased phagocytic rates in murine alveolar macrophages and phagocytic amoeba *D. discoideum*. The authors further showed that trypacidin reduced the viability of amoebae which signifies its role in conidial protection in the environment [105].

#### Fumagillin

Fumagillin (**9**) belongs to the meroterpenoid class of secondary metabolites. It was discovered in 1949 from *A. fumigatus* [127]. Strikingly, unlike other secondary metabolite synthesizing clusters, the fumagillin biosynthetic cluster is intertwined with the pseurotin gene cluster and designated as the *fma* cluster [95,128]. Wiemann and colleagues (2013) have shown the existence of a similarly intertwined pattern in both close and distant relatives of *A. fumigatus*, and therefore suggested a role of these metabolites in survival. In *A. fumigatus*, the *fma* cluster is located on the sub-telomeric region on chromosome 8 and is comprised of 15 genes. At the cellular level fumagillin is regulated by both cluster specific regulator FumR (FapR) and global regulator LaeA [95].

Fumagillin consists of a cyclohexane ring and decatetraenedioic acid connected via an ester bond. There is also a methoxy group, an epoxide and a terpene derived aliphatic chain that contains another epoxide, linked to cyclohexane. These unstable di-epoxides are responsible for the biological activity of fumagillin, which targets the active site of the methionine aminopeptidase type-2 (MetAP-2) enzyme [129]. MetAP-2 is involved in cell proliferation, translation and post-translational modifications of nascent polypeptides and is therefore essential for cell viability [130–131]. Additionally, fumagillin is also known to be overproduced upon caspofungin treatment and damage to the cell walls while fumagillin aids in immune evasion by reducing ROS levels, degranulation and actin filamentation in neutrophils [60,132]. In nature, several fungal species are known to produce caspofungin which could trigger fumagillin production in natural environments [132–133]. *A. fumigatus* possesses an additional MetAP-2 gene in the *fma* cluster that protects itself against its own toxin [134]. Fumagillin has therapeutic potential for the treatment of intestinal microsporidiosis and nosemiasis in honey bees [58,135]. Overall, antibiotic, immunosuppressive, antitumor and antiangiogenic properties have been attributed to fumagillin [129,136–140]. Specific antibiotic activities were demonstrated against the pathogen *Entamoeba histolytica* and later against eukaryotic parasites such as *Trypanosoma* and *Plasmodium* the causative agent of malaria [141–142]. In comparison to gliotoxin, we found only minor cytotoxic activities of fumagillin against the model amoebae *D. discoideum* [82]. It could still be conceivable that other amoeba could reveal higher sensitivity, but tests against the fungivorous amoeba *P. aurantium* were not yet conducted.

#### DHN-melanin

**Figure 5:**
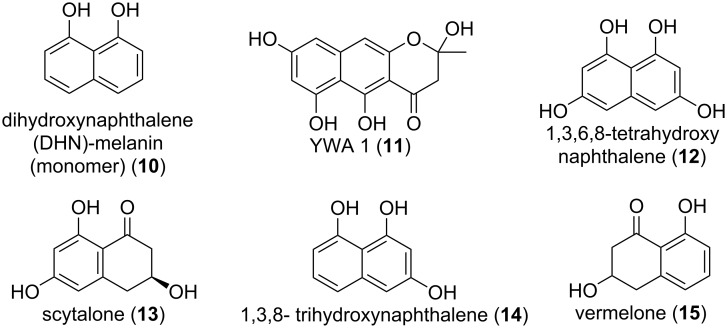
Intermediates of the DHN-melanin biosynthesis in *Aspergillus fumigatus*.

Melanins are a heterogenous group of hydrophobic phenolic polymers that are found in a range of organisms including bacteria, plants, fungi and even animals. The melanin pigments are of mostly dark colours like black or brown and are associated with virulence in plant- and animal-pathogenic fungi [143–145]. Three types of melanins are known to be produced by fungi of which *A. fumigatus* is able to produce two – pyomelanin and dihydroxynaphthalene melanin (DHN-melanin). While the water-soluble pyomelanin is synthesized via the tyrosine degradation pathway, the DHN-melanin synthesis relies on its own SM-gene-cluster [146–148]. The DHN-melanin of *A. fumigatus* is a heteropolymer formed through the polymerization of 1,8-dihydroxynaphtalene (1,8-DHN) monomers (**10**) and is responsible for the unique greyish-green colour of *A. fumigatus* conidia (Figure 5).

The genetics and biochemistry of its biosynthesis are well established: the 19 kb gene cluster contains 6 genes and lies downstream of the conidiation pathway. The polyketide synthase PksP combines the starter units acetyl-CoA and malonyl-CoA into the heptaketide naphthopyrone YWA1 (**11**). The hydrolytic activity of Ayg1 shortens the heptaketide to the pentaketide 1,3,6,8-tetrahydroxynaphthalene (1,3,6,8-THN) (**12**) and is further reduced by reductase Arp2 to scytalone (**13**), which in turn is dehydrated by Arp1 to 1,3,8-trihydroxynaphthalene (1,3,8-THN) (**14**). Again, Arp2 reduces 1,3,8-THN to vermelone (**15**) before it is dehydrated to 1,8-dihydroxynaphthalene (1,8-DHN) (**10**) by Abr1, a multi-copper reductase. In a last step polymerization of 1,8-DHN monomers is facilitated by the laccase Abr2 [45,149–152]. Knock out mutants of either *ayg1*, *arp2*, or *abr2* lead to different coloured conidia while loss of *pksP* aborts DHN-melanin synthesis completely which leads to white spores [45]. DHN-melanin is a heterogeneous polymer, as such it does not have a unique structure. Its insolubility aggravates any structural analyses of the deciphering of repetitive motives. However, there were studies doing either computational predictions or artificial oxidative polymerization studies of 1,8-DHN monomers [144,153].

Next to offering the conidia protection from UV radiation, DHN-melanin was shown to be a key factor to survival during both predation and virulence. When preyed upon by fungivorous amoeba like *P. aurantium* melanised conidia where not only internalized less than Δ*pksP* conidia but were also able to prevent maturation of phagolysosomes [50,147]. During infection DHN-melanin masks the pathogen-associated molecular patterns on the spore-surface and is thus less likely to be recognized by the immune system. The Δ*pksP* strain lacks this protection and is more easily recognized by the immune system, thus triggering a stronger immune response, including a higher pro-inflammatory response and increased recognition and ingestion by phagocytes rendering the Δ*pksP* strain less virulent. Additionally, melanised conidia are more likely to survive internalization by lung epithelial cells [147,154–155]. Although DHN-melanin is generally associated with immune evasion it was recently found to be recognized in higher animals via the C-type lectin receptor (MelLec) which interacts with the naphthalene-diol domain of DHN-melanin. Additionally, the surfactant protein D (SP-D), a soluble C-type lectin receptor (CLR), is also able to recognise DHN-melanin and opsonize it to increase the immune response. However, MelLec receptors are only present on some endothelial and myeloid cells [156–157].

#### Fumigaclavines

**Figure 6:**
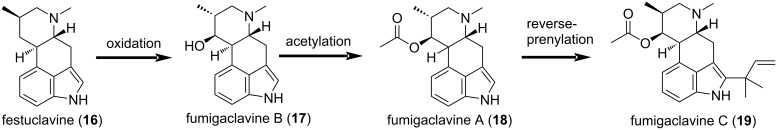
Intermediates and products of the fumigaclavine C biosynthesis.

Fumigaclavine C (**19**) is a tryptophan-derived indole alkaloid which was so far only shown to be produced by *A. fumigatus* while other fumigaclavines can for example also be found in *Penicillium* ssp. (fumigaclavine A (**18**) and B (**17**)) [66,158]. In all fungi, alkaloid biosynthetic pathways share a common basis, starting with the prenylation of ʟ-tryptophan to dimethylallyltryptophan (DMAT). During several steps DMAT is converted to chanoclavine-I aldehyde, the last mutual intermediate. Branching into different pathways after this intermediate is mainly due to differences in the function of EasA, the enzyme catalysing the next biosynthetic step. In *A. fumigatus* EasA acts as a reductase and after additional steps chanoclavine-I aldehyde is converted into festuclavine (**16**) (Figure 6). Festuclavine is then oxidized to fumigaclavine B (**17**) which in turn is acetylated to fumigaclavine A (**18**). Finally a reverse prenylation of fumigaclavine A leads to fumigaclavine C (**19**), the final product of fumigaclavine biosynthesis [159]. Biosynthesis of the intermediate festuclavine as well as fumigaclavines A–C is dependent on LaeA regulation [124].

Its numerous bioactive effects hold the potential for a pharmaceutical use since it was shown to be an effective inhibitor of tumor necrosis factor-alpha (TNF-α) production by preventing the activation of TLR4 by lipopolysaccharide (LPS) and was thus proposed for potential use against atherosclerosis [67]. Furthermore fumigaclavine C has also proven effective against MCF-7 breast cancer cells by arresting the cell cycle and promoting apoptosis while showing no cytotoxicity against RAW 264.7 cells, thus demonstrating their selectivity [65,67]. Further, fumigaclavine was shown to exhibit antibacterial properties and to contribute to virulence in the model insect *Galleria mellonella* [66].

#### Fumitremorgins

**Figure 7:**
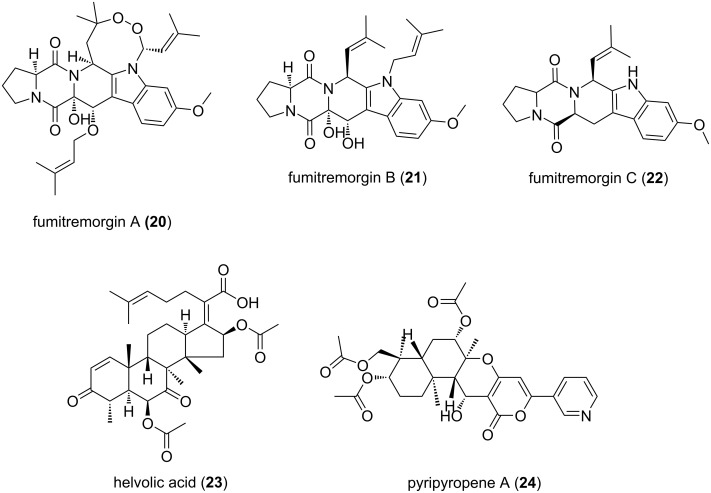
Bioactive secondary metabolites of *Aspergillus fumigatus*.

The class of fumitremorgins comprises several diketopiperazine alkaloids which are tremorgenic mycotoxins. However, there are several fumitremorgin-like indole alkaloids including tryprostatins, spiro- and cyclotryprostatins and verruculogen besides fumitremorgins themselves. They occur most often in *Aspergillus* and *Penicillium* species [160]. Fumitremorgin A (**20**), B (**21**) and C (**22**) can all be found in *A. fumigatus* (Figure 7). They are based on the precursers ʟ-tryptophan and ʟ-proline and are further derived from breviamide F, proposedly via tryprostatin B which is hydroxylated and methylated to tryprostatin A. Oxidative closure of the ringstructure then results in fumitremorgin C. Further modification of the structure leads to fumitremorgin B and verruculogen, which shares the same pathway [97,160–162]. Which enzyme is responsible for the conversion of verruculogen to fumitremorgin A remains to be elucidated. Like several other clusters, the biosynthesis of fumitremorgins is dependent on LaeA [124].

Fumitremorgin B was shown to have antifungal properties against phytopathogenic fungi, antifeedant properties against army-worm larvae and toxic on brine shrimp [72]. It was further shown to be cytotoxic and inhibiting cell cycle progression at G2/M phase [163]. Fumitremorgin C was shown to effect mammalian cells and inhibit the breast cancer resistance protein which imparts multidrug resistance and thus resistance to chemotherapeutics in breast cancer treatment [75,164].

#### Helvolic acid

**Figure 8:**
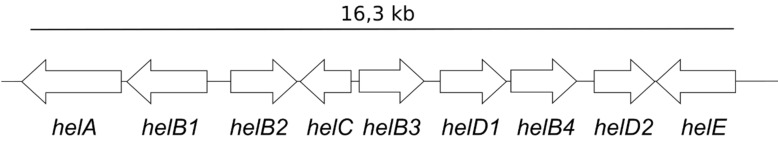
Helvolic acid gene cluster of *A. fumigatus.*

Helvolic acid (HA) (**23**) is a fusidane-type antibiotic that belongs to the triterpenoid class of secondary metabolites. Originally, it was discovered from *A. fumigatus* but later several other members of the sub phylum Pezizomycotina were also found to be HA producers [165–168]. In *A. fumigatus*, the biosynthetic cluster of HA is comprised of 9 genes that spans over a 16.3 kb region on chromosome 4 (Figure 8). The cluster contains an oxidosqualine cyclase (*helA*), three Cytochrome P450 (*helB1*, *helB2*, *helB3*), a short-chain dehydrogenase/reductase (*helC*) and two acetyltransferases (*helD1*, *helD2*) and a 3-ketosteroid-Δ^1^-dehydrogenase [83,169]. Helvolic acid is a tetracyclic compound containing two keto groups, two acetates and one carboxyl group which do not equally contribute to function [169]. Lv and colleagues have shown that the presence of both the C-20 carboxyl group and the 3-keto group are crucial for its antibacterial activity whereas, acetylation of the C-6 hydroxy group reduces the activity of HA [169]. Previous studies have also shown the antitrypanosomal, antifungal and cilioinhibitory properties of HA [72,83,86–88]. For these properties and little cross-resistance helvolic acid is of great pharmaceutical importance. On the other hand, these antibiotic activities of HA could alter the soil microflora in natural habitats, an ecological role of HA that requires further investigation.

#### Pyripyropene A

Pyripyropene A (PPPA) (**24**) belongs to the meroterpenoid class of secondary metabolites. It was originally isolated from *A. fumigatus*, but later several other pyripyropene A producing members of *Aspergillus* and *Penicillium* ssp. were identified [98–99,170–171]. In A. fumigatus 9 genes form a pyripyropene A (pyr) biosynthetic cluster that spans a 23 kb region on chromosome 6 [172]. Chemically, pyripyropene (PP) analogs are meroterpenoids containing a fused pyridyl α-pyrone moiety and eight contiguous stereocenters [170]. Metabolically, PPPA non-covalently binds within the fifth transmembrane domain of acyl-coenzyme A (CoA):cholesterol acyltransferase ACAT2 and renders it inactive [173]. In vivo, PPPA-mediated ACAT2 inhibition was shown to protect the mice from atherosclerosis, ACAT2 enzyme mediates in lipid metabolism and is localized in the liver and intestines [174]. Furthermore, PPPA was also shown to exhibit insecticidal properties against aphids [100].

## Conclusion

Increasing access to sequenced microbial genomes offers a glimpse at the untapped potential we have yet to gain access to. Fungi in particular harbor great potential to produce novel secondary metabolites with ecological and pathogenic importance. As a medically relevant fungal pathogen *A. fumigatus* is the subject of much research and since sequencing of its genome in 2005 its potential for the production of secondary metabolites was scrutinized frequently [7,43,175]. In recent years many of its BGCs could be matched with either long known or newly discovered bioactive compounds and while the bioactive potential and the ecological role of many well studied metabolites like DHN-melanin or gliotoxin is well known, newer metabolites often cannot be associated with a biological function. Due to its clinical significance, the highest interest in secondary metabolites of *A. fumigatus* was driven by its pathobiology, e.g., a role in cytotoxicity, immunosuppression or antifungal drug resistance. In natural habitats these molecules may fulfill analogous functions, such as the defense against phagocytic predators by gliotoxin [78–82]. Indeed, the need for survival is the driving force of evolution and fungi like *A. fumigatus* were able to cultivate an impressive arsenal of protective mechanism from DHN-melanin which offers mostly passive protection to more active compounds like fumigaclavines or helvolic acid with their antibacterial and antifungal activities, respectively [50,63,72,147]. Since SM activities are most often closely related to ecological conditions mimicking of more natural cultivation conditions might lead to the discovery of new compounds and their ecological role.

In the past few years, protists like *D. discoideum* and *Acanthamoeba castellani* have been widely used for the identification of virulence attributes of pathogenic fungi, including *Aspergillus* spp., for their similarity with human phagocytic cells [32]. Nevertheless, the precise identity of amoeboid, nematode and arthropod predators that target filamentous fungi in their environmental niches remained elusive and has been limited by their biological complexity. It was thus surprising to find that the environmentally abundant, fungivorous amoeba *P. aurantium* does not only graze on yeast but can specifically target filamentous fungi such as *A. fumigatus*. The mechanism of action was coined ruphocytosis and involved a locally distinct disruption of the cell wall of the fungal hyphae to feed on the fungal cytoplasm [29]. It is well conceivable that this amoeba will target a range of different filamentous fungi, and that this biotic cell wall stress can be exploited as an ecological trigger for the production and identification of new bioactive compounds in the future.

## References

[1] Blackwell M (2011). Am J Bot.

[2] Cantrell S A, Dianese J C, Fell J, Gunde-Cimerman N, Zalar P (2011). Mycologia.

[3] Sutherland I W (2001). Trends Microbiol.

[4] Costa-Orlandi C B, Sardi J C O, Pitangui N S, de Oliveira H C, Scorzoni L, Galeane M C, Medina-Alarcón K P, Melo W, Marcelino M Y, Braz J D (2017). J Fungi.

[5] Kowalski C H, Morelli K A, Schultz D, Nadell C D, Cramer R A (2020). Proc Natl Acad Sci U S A.

[6] Spiteller P (2015). Nat Prod Rep.

[7] Nierman W C, Pain A, Anderson M J, Wortman J R, Kim H S, Arroyo J, Berriman M, Abe K, Archer D B, Bermejo C (2005). Nature.

[8] Khaldi N, Seifuddin F T, Turner G, Haft D, Nierman W C, Wolfe K H, Fedorova N D (2010). Fungal Genet Biol.

[9] Brakhage A A, Schroeckh V (2011). Fungal Genet Biol.

[10] Keller N P, Turner G, Bennett J W (2005). Nat Rev Microbiol.

[11] Gacek A, Strauss J (2012). Appl Microbiol Biotechnol.

[12] Krause D J, Kominek J, Opulente D A, Shen X-X, Zhou X, Langdon Q K, DeVirgilio J, Hulfachor A B, Kurtzman C P, Rokas A (2018). Proc Natl Acad Sci U S A.

[13] Smith D J, Burnham M K R, Edwards J, Earl A J, Turner G (1990). Bio/Technology.

[14] Keller N P (2019). Nat Rev Microbiol.

[15] O'Donnell K, Cigelnik E, Casper H H (1998). Fungal Genet Biol.

[16] Martin F, Kohler A, Murat C, Balestrini R, Coutinho P M, Jaillon O, Montanini B, Morin E, Noel B, Percudani R (2010). Nature.

[17] Karwehl S, Stadler M, Stadler M, Dersch P (2016). Exploitation of Fungal Biodiversity for Discovery of Novel Antibiotics. How to Overcome the Antibiotic Crisis : Facts, Challenges, Technologies and Future Perspectives.

[18] Spiteller P (2008). Chem – Eur J.

[19] Zeng R S L, Niu G, Wen Z, Schuler M A, Berenbaum M R (2006). J Chem Ecol.

[20] González-Osnaya L, Soriano J M, Moltó J C, Mañes J (2007). Food Addit Contam.

[21] Reiss J (1975). Chem-Biol Interact.

[22] Cary J W, Harris-Coward P Y, Ehrlich K C, Di Mavungu J D, Malysheva S V, De Saeger S, Dowd P F, Shantappa S, Martens S L, Calvo A M (2014). Fungal Genet Biol.

[23] Zhao Y, Ding J, Yuan W, Huang J, Huang W, Wang Y, Zheng W (2017). Environ Microbiol.

[24] Xu Y, Vinas M, Alsarrag A, Su L, Pfohl K, Rohlfs M, Schäfer W, Chen W, Karlovsky P (2019). Nat Commun.

[25] Eadie M J (2003). Lancet Neurol.

[26] Tudzynski P, Correia T, Keller U (2001). Appl Microbiol Biotechnol.

[27] Tudzynski P, Scheffer J (2004). Mol Plant Pathol.

[28] Schardl C L, Panaccione D G, Tudzynski P (2006). Ergot Alkaloids – Biology and Molecular Biology. The Alkaloids: Chemistry and Biology.

[29] Radosa S, Ferling I, Sprague J L, Westermann M, Hillmann F (2019). Environ Microbiol.

[30] Van Waeyenberghe L, Baré J, Pasmans F, Claeys M, Bert W, Haesebrouck F, Houf K, Martel A (2013). Environ Microbiol Rep.

[31] Brunke S, Mogavero S, Kasper L, Hube B (2016). Curr Opin Microbiol.

[32] Tosetti N, Croxatto A, Greub G (2014). Microb Pathog.

[33] Sanchez J F, Somoza A D, Keller N P, Wang C C C (2012). Nat Prod Rep.

[34] Show P L, Oladele K O, Siew Q Y, Aziz Zakry F A, Lan J C-W, Ling T C (2015). Front Life Sci.

[35] Stötefeld L, Scheu S, Rohlfs M (2012). Ecol Entomol.

[36] Rohlfs M, Zeilinger S, Martín J F, García-Estrada C (2015). Fungal secondary metabolism in the light of animal–fungus interactions: from mechanism to ecological function. Biosynthesis and Molecular Genetics of Fungal Secondary Metabolites.

[37] Künzler M (2018). PLoS Pathog.

[38] Brakhage A A, Langfelder K (2002). Annu Rev Microbiol.

[39] Novohradská S, Ferling I, Hillmann F (2017). Front Cell Infect Microbiol.

[40] Latgé J-P (1999). Clin Microbiol Rev.

[41] Bignell E, Cairns T C, Throckmorton K, Nierman W C, Keller N P (2016). Philos Trans R Soc, B.

[42] Frisvad J C, Rank C, Nielsen K F, Larsen T O (2009). Med Mycol.

[43] Inglis D O, Binkley J, Skrzypek M S, Arnaud M B, Cerqueira G C, Shah P, Wymore F, Wortman J R, Sherlock G (2013). BMC Microbiol.

[44] Lind A L, Wisecaver J H, Lameiras C, Wiemann P, Palmer J M, Keller N P, Rodrigues F, Goldman G H, Rokas A (2017). PLoS Biol.

[45] Heinekamp T, Thywißen A, Macheleidt J, Keller S, Valiante V, Brakhage A A (2013). Front Microbiol.

[46] Gómez B L, Nosanchuk J D (2003). Curr Opin Infect Dis.

[47] Nguyen K-H, Chollet-Krugler M, Gouault N, Tomasi S (2013). Nat Prod Rep.

[48] Brakhage A A, Liebmann B (2005). Med Mycol.

[49] Jahn B, Langfelder K, Schneider U, Schindel C, Brakhage A A (2002). Cell Microbiol.

[50] Ferling I, Dunn J D, Ferling A, Soldati T, Hillmann F (2020). mBio.

[51] Lim F Y, Hou Y, Chen Y, Oh J-H, Lee I, Bugni T S, Keller N P (2012). Appl Environ Microbiol.

[52] Berthier E, Lim F Y, Deng Q, Guo C-J, Kontoyiannis D P, Wang C C C, Rindy J, Beebe D J, Huttenlocher A, Keller N P (2013). PLoS Pathog.

[53] Throckmorton K, Lim F Y, Kontoyiannis D P, Zheng W, Keller N P (2016). Environ Microbiol.

[54] Hissen A H T, Wan A N C, Warwas M L, Pinto L J, Moore M M (2005). Infect Immun.

[55] Schrettl M, Bignell E, Kragl C, Joechl C, Rogers T, Arst H N, Haynes K, Haas H (2004). J Exp Med.

[56] Kato N, Suzuki H, Okumura H, Takahashi S, Osada H (2013). Biosci, Biotechnol, Biochem.

[57] Ehlers T, Furness S, Robinson T P, Zhong H A, Goldsmith D, Aribser J, Bowen J P (2016). Curr Top Med Chem.

[58] van den Heever J P, Thompson T S, Curtis J M, Ibrahim A, Pernal S F (2014). J Agric Food Chem.

[59] Mendoza Y, Diaz-Cetti S, Ramallo G, Santos E, Porrini M, Invernizzi C (2017). J Econ Entomol.

[60] Fallon J P, Reeves E P, Kavanagh K (2010). J Med Microbiol.

[61] Fallon J P, Reeves E P, Kavanagh K (2011). Microbiology (Reading, U K).

[62] Guruceaga X, Ezpeleta G, Mayayo E, Sueiro-Olivares M, Abad-Diaz-De-Cerio A, Aguirre Urízar J M, Liu H G, Wiemann P, Bok J W, Filler S G (2018). Virulence.

[63] Pinheiro E A A, Carvalho J M, dos Santos D C P, Feitosa A d O, Marinho P S B, Guilhon G M S P, de Souza A D L, da Silva F M A, Marinho A M d R (2013). Nat Prod Res.

[64] Ma H-Y, Song Y-C, Mao Y-Y, Jiang J-H, Tan R-X, Luo L (2006). Planta Med.

[65] Li Y-X, Himaya S W A, Dewapriya P, Zhang C, Kim S-K (2013). Mar Drugs.

[66] Panaccione D G, Arnold S L (2017). Sci Rep.

[67] Du R H, Li E G, Cao Y, Song Y C, Tan R X (2011). Life Sci.

[68] Macheleidt J, Scherlach K, Neuwirth T, Schmidt-Heck W, Straßburger M, Spraker J, Baccile J A, Schroeder F C, Keller N P, Hertweck C (2015). Mol Microbiol.

[69] Lim F Y, Ames B, Walsh C T, Keller N P (2014). Cell Microbiol.

[70] Garcia Silva M, Araçari Jacometti Cardoso Furtado N, Tallarico Pupo M, José Vieira Fonseca M, Said S, Alves da Silva Filho A, Kenupp Bastos J (2004). Microbiol Res.

[71] Belofsky G N, Anguera M, Jensen P R, Fenical W, Köck M (2000). Chem – Eur J.

[72] Li X-J, Zhang Q, Zhang A-L, Gao J-M (2012). J Agric Food Chem.

[73] Baccile J A, Spraker J E, Le H H, Brandenburger E, Gomez C, Bok J W, Macheleidt J, Brakhage A A, Hoffmeister D, Keller N P (2016). Nat Chem Biol.

[74] Khalid S, Baccile J A, Spraker J E, Tannous J, Imran M, Schroeder F C, Keller N P (2018). ACS Chem Biol.

[75] González-Lobato L, Real R, Prieto J G, Álvarez A I, Merino G (2010). Eur J Pharmacol.

[76] Lehner S M, Atanasova L, Neumann N K N, Krska R, Lemmens M, Druzhinina I S, Schuhmacher R (2013). Appl Environ Microbiol.

[77] Ali H, Ries M I, Lankhorst P P, van der Hoeven R A M, Schouten O L, Noga M, Hankemeier T, van Peij N N M E, Bovenberg R A L, Vreeken R J (2014). PLoS One.

[78] Scharf D H, Brakhage A A, Mukherjee P K (2016). Environ Microbiol.

[79] Yamada A, Kataoka T, Nagai K (2000). Immunol Lett.

[80] Schlam D, Canton J, Carreño M, Kopinski H, Freeman S A, Grinstein S, Fairn G D (2016). mBio.

[81] Gardiner D M, Waring P, Howlett B J (2005). Microbiology (Reading, U K).

[82] Hillmann F, Novohradská S, Mattern D J, Forberger T, Heinekamp T, Westermann M, Winckler T, Brakhage A A (2015). Environ Microbiol.

[83] Mitsuguchi H, Seshime Y, Fujii I, Shibuya M, Ebizuka Y, Kushiro T (2009). J Am Chem Soc.

[84] Lodeiro S, Xiong Q, Wilson W K, Ivanova Y, Smith M L, May G S, Matsuda S P T (2009). Org Lett.

[85] Kimura M, Kushiro T, Shibuya M, Ebizuka Y, Abe I (2010). Biochem Biophys Res Commun.

[86] Ganaha M, Yoshii K, Ōtsuki Y, Iguchi M, Okamoto Y, Iseki K, Ban S, Ishiyama A, Hokari R, Iwatsuki M (2016). Chem Pharm Bull.

[87] Amitani R, Taylor G, Elezis E-N, Llewellyn-Jones C, Mitchell J, Kuze F, Cole P J, Wilson R (1995). Infect Immun.

[88] Kong F-D, Huang X-L, Ma Q-Y, Xie Q-Y, Wang P, Chen P-W, Zhou L-M, Yuan J-Z, Dai H-F, Luo D-Q (2018). J Nat Prod.

[89] Yin W-B, Baccile J A, Bok J W, Chen Y, Keller N P, Schroeder F C (2013). J Am Chem Soc.

[90] Wiemann P, Lechner B E, Baccile J A, Velk T A, Yin W-B, Bok J W, Pakala S, Losada L, Nierman W C, Schroeder F C (2014). Front Microbiol.

[91] König C C, Scherlach K, Schroeckh V, Horn F, Nietzsche S, Brakhage A A, Hertweck C (2013). ChemBioChem.

[92] Chooi Y-H, Fang J, Liu H, Filler S G, Wang P, Tang Y (2013). Org Lett.

[93] Andersen M R, Nielsen J B, Klitgaard A, Petersen L M, Zachariasen M, Hansen T J, Blicher L H, Gotfredsen C H, Larsen T O, Nielsen K F (2013). Proc Natl Acad Sci U S A.

[94] Mehedi M A U, Molla A H, Khondkar P, Sultana S, Islam M A, Rashid M A, Chowdhury R (2010). Asian J Chem.

[95] Wiemann P, Guo C-J, Palmer J M, Sekonyela R, Wang C C C, Keller N P (2013). Proc Natl Acad Sci U S A.

[96] Ishikawa M, Ninomiya T, Akabane H, Kushida N, Tsujiuchi G, Ohyama M, Gomi S, Shito K, Murata T (2009). Bioorg Med Chem Lett.

[97] Maiya S, Grundmann A, Li X, Li S-M, Turner G (2007). ChemBioChem.

[98] Omura S, Tomoda H, Kim Y K, Nishida H (1993). J Antibiot.

[99] Hu J, Furutani A, Yamamoto K, Oyama K, Mitomi M, Anzai H (2014). Biotechnol Biotechnol Equip.

[100] Goto K, Horikoshi R, Mitomi M, Oyama K, Hirose T, Sunazuka T, Ōmura S (2018). J Antibiot.

[101] VanMiddlesworth F, Dufresne C, Wincott F E, Mosley R T, Wilson K E (1992). Tetrahedron Lett.

[102] VanMiddlesworth F, Giacobbe R A, Lopez M, Garrity G, Bland J A, Bartizal K, Fromtling R A, Polishook J, Zweerink M, Edison A M (1992). J Antibiot.

[103] Rank C (2010). Mapping of secondary metabolism in biotechnologically important Aspergillus species.

[104] Kobayashi S, Furuta T, Hayashi T, Nishijima M, Hanada K (1998). J Am Chem Soc.

[105] Mattern D J, Schoeler H, Weber J, Novohradská S, Kraibooj K, Dahse H-M, Hillmann F, Valiante V, Figge M T, Brakhage A A (2015). Appl Microbiol Biotechnol.

[106] Balan J, Ebringer L, Nemec P, Kováč Š, Dobias J (1963). J Antibiot, Ser A.

[107] Gauthier T, Wang X, Sifuentes Dos Santos J, Fysikopoulos A, Tadrist S, Canlet C, Artigot M P, Loiseau N, Oswald I P, Puel O (2012). PLoS One.

[108] Khoufache K, Puel O, Loiseau N, Delaforge M, Rivollet D, Coste A, Cordonnier C, Escudier E, Botterel F, Bretagne S (2007). BMC Microbiol.

[109] Gallagher R T, Latch G C M (1977). Appl Environ Microbiol.

[110] Wang F, Fang Y, Zhu T, Zhang M, Lin A, Gu Q, Zhu W (2008). Tetrahedron.

[111] Lim F Y, Won T H, Raffa N, Baccile J A, Wisecaver J, Rokas A, Schroeder F C, Keller N P (2018). mBio.

[112] Bok J W, Chung D, Balajee S A, Marr K A, Andes D, Nielsen K F, Frisvad J C, Kirby K A, Keller N P (2006). Infect Immun.

[113] Bell M R, Johnson J R, Wildi B S, Woodward R B (1958). J Am Chem Soc.

[114] Bok J W, Balajee S A, Marr K A, Andes D, Nielsen K F, Frisvad J C, Keller N P (2005). Eukaryotic Cell.

[115] Cramer R A, Gamcsik M P, Brooking R M, Najvar L K, Kirkpatrick W R, Patterson T F, Balibar C J, Graybill J R, Perfect J R, Abraham S N (2006). Eukaryotic Cell.

[116] Kupfahl C, Heinekamp T, Geginat G, Ruppert T, Härtl A, Hof H, Brakhage A A (2006). Mol Microbiol.

[117] Scharf D H, Remme N, Heinekamp T, Hortschansky P, Brakhage A A, Hertweck C (2010). J Am Chem Soc.

[118] Schrettl M, Carberry S, Kavanagh K, Haas H, Jones G W, O'Brien J, Nolan A, Stephens J, Fenelon O, Doyle S (2010). PLoS Pathog.

[119] McDonagh A, Fedorova N D, Crabtree J, Yu Y, Kim S, Chen D, Loss O, Cairns T, Goldman G, Armstrong-James D (2008). PLoS Pathog.

[120] Sugui J A, Kim H S, Zarember K A, Chang Y C, Gallin J I, Nierman W C, Kwon-Chung K J (2008). PLoS One.

[121] Sugui J A, Pardo J, Chang Y C, Zarember K A, Nardone G, Galvez E M, Müllbacher A, Gallin J I, Simon M M, Kwon-Chung K J (2007). Eukaryotic Cell.

[122] Vargas W A, Mukherjee P K, Laughlin D, Wiest A, Moran-Diez M E, Kenerley C M (2014). Microbiology (Reading, U K).

[123] Coyle C M, Kenaley S C, Rittenour W R, Panaccione D G (2007). Mycologia.

[124] Perrin R M, Fedorova N D, Bok J W, Cramer R A, Wortman J R, Kim H S, Nierman W C, Keller N P (2007). PLoS Pathog.

[125] Twumasi-Boateng K, Yu Y, Chen D, Gravelat F N, Nierman W C, Sheppard D C (2009). Eukaryotic Cell.

[126] Upadhyay S, Torres G, Lin X (2013). Eukaryotic Cell.

[127] Hanson F R, Eble T E (1949). J Bacteriol.

[128] Lin H-C, Chooi Y-H, Dhingra S, Xu W, Calvo A M, Tang Y (2013). J Am Chem Soc.

[129] Sin N, Meng L, Wang M Q W, Wen J J, Bornmann W G, Crews C M (1997). Proc Natl Acad Sci U S A.

[130] Mauriz J L, Martín-Renedo J, García-Palomo A, Tuñón M J, González-Gallego J (2010). Curr Drug Targets.

[131] Vetro J A, Dummitt B, Chang Y-H, Hooper N M, Lendeckel U (2004). Methionine Aminopeptidase. Aminopeptidases in Biology and Disease.

[132] Conrad T, Kniemeyer O, Henkel S G, Krüger T, Mattern D J, Valiante V, Guthke R, Jacobsen I D, Brakhage A A, Vlaic S (2018). BMC Syst Biol.

[133] Netzker T, Fischer J, Weber J, Mattern D J, König C C, Valiante V, Schroeckh V, Brakhage A A (2015). Front Microbiol.

[134] Guruceaga X, Perez-Cuesta U, Abad-Diaz de Cerio A, Gonzalez O, Alonso R M, Hernando F L, Ramirez-Garcia A, Rementeria A (2019). Toxins.

[135] Molina J-M, Tourneur M, Sarfati C, Chevret S, de Gouvello A, Gobert J-G, Balkan S, Derouin F (2002). N Engl J Med.

[136] Keller N, Bok J, Chung D, Perrin R M, Keats Shwab E (2006). Med Mycol.

[137] Romsdahl J, Wang C C C (2019). Med Chem Commun.

[138] Wang J, Sheppard G S, Lou P, Kawai M, BaMaung N, Erickson S A, Tucker-Garcia L, Park C, Bouska J, Wang Y-C (2003). Cancer Res.

[139] Yamaoka M, Yamamoto T, Ikeyama S, Sudo K, Fujita T (1993). Cancer Res.

[140] Tucker L A, Zhang Q, Sheppard G S, Lou P, Jiang F, McKeegan E, Lesniewski R, Davidsen S K, Bell R L, Wang J (2008). Oncogene.

[141] McCowen M C, Callender M E, Lawlis J F (1951). Science.

[142] Arico-Muendel C, Centrella P A, Contonio B D, Morgan B A, O’Donovan G, Paradise C L, Skinner S R, Sluboski B, Svendsen J L, White K F (2009). Bioorg Med Chem Lett.

[143] Eisenman H C, Casadevall A (2012). Appl Microbiol Biotechnol.

[144] Cecchini M M, Reale S, Manini P, d'Ischia M, De Angelis F (2017). Chem – Eur J.

[145] Tsai H-F, Washburn R G, Chang Y C, Kwon‐Chung K J (1997). Mol Microbiol.

[146] Schmaler-Ripcke J, Sugareva V, Gebhardt P, Winkler R, Kniemeyer O, Heinekamp T, Brakhage A A (2009). Appl Environ Microbiol.

[147] Perez-Cuesta U, Aparicio-Fernandez L, Guruceaga X, Martin-Souto L, Abad-Diaz-de-Cerio A, Antoran A, Buldain I, Hernando F L, Ramirez-Garcia A, Rementeria A (2020). Int Microbiol.

[148] Tsai H-F, Wheeler M H, Chang Y C, Kwon-Chung K J (1999). J Bacteriol.

[149] Fujii I, Yasuoka Y, Tsai H-F, Chang Y C, Kwon-Chung K J, Ebizuka Y (2004). J Biol Chem.

[150] Langfelder K, Jahn B, Gehringer H, Schmidt A, Wanner G, Brakhage A A (1998). Med Microbiol Immunol.

[151] Tsai H-F, Fujii I, Watanabe A, Wheeler M H, Chang Y C, Yasuoka Y, Ebizuka Y, Kwon-Chung K J (2001). J Biol Chem.

[152] Sugareva V, Härtl A, Brock M, Hübner K, Rohde M, Heinekamp T, Brakhage A A (2006). Arch Microbiol.

[153] Manini P, Bietti M, Galeotti M, Salamone M, Lanzalunga O, Cecchini M M, Reale S, Crescenzi O, Napolitano A, De Angelis F (2018). ACS Omega.

[154] Chai L Y A, Netea M G, Sugui J, Vonk A G, van de Sande W W J, Warris A, Kwon-Chung K J, Kullberg B J (2010). Immunobiology.

[155] Thywißen A, Heinekamp T, Dahse H-M, Schmaler-Ripcke J, Nietsche S, Zipfel P F, Brakhage A A (2011). Front Microbiol.

[156] Stappers M H T, Clark A E, Aimanianda V, Bidula S, Reid D M, Asamaphan P, Hardison S E, Dambuza I M, Valsecchi I, Kerscher B (2018). Nature.

[157] Wong S S W, Rani M, Dodagatta-Marri E, Ibrahim-Granet O, Kishore U, Bayry J, Latgé J-P, Sahu A, Madan T, Aimanianda V (2018). J Biol Chem.

[158] Kozlovskii A G, Zhelifonova V P, Antipova T V (2013). Appl Biochem Microbiol.

[159] Robinson S L, Panaccione D G (2015). Toxins.

[160] Li S-M (2011). J Antibiot.

[161] Grundmann A, Li S-M (2005). Microbiology (Reading, U K).

[162] Yamazaki M, Fujimoto H, Kawasaki T (1980). Chem Pharm Bull.

[163] Cui C-B, Kakeya H, Osada H (1997). Tetrahedron.

[164] Rabindran S K, Ross D D, Doyle L A, Yang W, Greenberger L M (2000). Cancer Res.

[165] Chain E, Florey H, Jennings M, Williams T (1943). Br J Exp Pathol.

[166] Rank C, Larsen T O, Frisvad J C, Machida M, Gomi K (2010). Functional systems biology of Aspergillus. Aspergillus: molecular biology and genomics.

[167] Tamiya H, Ochiai E, Kikuchi K, Yahiro M, Toyotome T, Watanabe A, Yaguchi T, Kamei K (2015). J Infect Chemother.

[168] Cole R J, Jarvis B B, Schweikert M A (2003). Handbook of secondary fungal metabolites.

[169] Lv J-M, Hu D, Gao H, Kushiro T, Awakawa T, Chen G-D, Wang C-X, Abe I, Yao X-S (2017). Nat Commun.

[170] Tomoda H, Nishida H, Kim Y K, Obata R, Sunazuka T, Omura S, Bordner J, Guadliana M, Dormer P G, Smith A B (1994). J Am Chem Soc.

[171] Kim Y K, Tomoda H, Nishida H, Sunazuka T, Obata R, Omura S (1994). J Antibiot.

[172] Itoh T, Tokunaga K, Matsuda Y, Fujii I, Abe I, Ebizuka Y, Kushiro T (2010). Nat Chem.

[173] Das A, Davis M A, Tomoda H, Ômura S, Rudel L L (2008). J Biol Chem.

[174] Ohshiro T, Matsuda D, Sakai K, Degirolamo C, Yagyu H, Rudel L L, Ōmura S, Ishibashi S, Tomoda H (2011). Arterioscler, Thromb, Vasc Biol.

[175] Raffa N, Keller N P (2019). PLoS Pathog.

